# Transcription Factor EB: A Promising Therapeutic Target for Ischemic Stroke

**DOI:** 10.2174/1570159X21666230724095558

**Published:** 2023-08-03

**Authors:** Jie Shao, Yue Lang, Manqiu Ding, Xiang Yin, Li Cui

**Affiliations:** 1 Department of Neurology and Neuroscience Center, The First Hospital of Jilin University, Jilin University, Changchun, China

**Keywords:** Ischemic stroke, transcription factor EB, lysosome, autophagy, therapeutic target, metabolism

## Abstract

Transcription factor EB (TFEB) is an important endogenous defensive protein that responds to ischemic stimuli. Acute ischemic stroke is a growing concern due to its high morbidity and mortality. Most survivors suffer from disabilities such as numbness or weakness in an arm or leg, facial droop, difficulty speaking or understanding speech, confusion, impaired balance or coordination, or loss of vision. Although TFEB plays a neuroprotective role, its potential effect on ischemic stroke remains unclear. This article describes the basic structure, regulation of transcriptional activity, and biological roles of TFEB relevant to ischemic stroke. Additionally, we explore the effects of TFEB on the various pathological processes underlying ischemic stroke and current therapeutic approaches. The information compiled here may inform clinical and basic studies on TFEB, which may be an effective therapeutic drug target for ischemic stroke.

## 1 INTRODUCTION

Acute ischemic stroke (AIS) occurs when a cerebral artery is blocked by a locally formed clot or by a clot mobilized in the blood or when global ischemia further reduces the blood supply to an already narrowed cerebral artery, resulting in damage to brain cells due to ischemia and hypoxia [1]. Given its high morbidity, disability, and mortality, AIS is considered a leading cause of preventable death worldwide [2]. The current clinical treatment for ischemic stroke remains to restore blood flow through pharmacological thrombolysis or mechanical thrombectomy, but the strict therapeutic time window and several contraindications limit both. It is estimated that only 11% of patients with ischemic stroke can receive intervention in the form of recombinant tissue plasminogen activator (alteplase), and almost half of these patients fail to show improvement [1, 3]. Furthermore, given the complexity of the mechanisms underlying cerebrovascular disease, the known neuroprotective agents cannot effectively treat stroke in humans [4]. Therefore, we wish to illustrate potentially tractable pharmacological targets by reviewing our current understanding of the pathogenesis of ischemic stroke.

Transcription factor EB (TFEB) belongs to the microphthalmia (MiT/TFE) family, which contains the basic helix-loop-helix leucine-zipper motif (bHLH-Zip) [5, 6]. TFEB is crucially involved in lysosomal biogenesis [7], lysosomal exocytosis [8], autophagy [9], lipid catabolism [10], energy metabolism [11], angiogenesis [12], and the immune response [13]. Furthermore, TFEB is protective against myocardial ischemia [14], inflammatory responses [13], and atherosclerosis [15]. TFEB has confirmed positive effects on neurodegenerative diseases [16], tumors [17], and kidney-related diseases [18]; however, its effects on AIS remain unclear.

In this article, we first introduce the background on TFEB and ischemic stroke. We then describe the regulatory mechanisms underlying the transcriptional activity of TFEB as well as its sensing and regulation of energy metabolism following ischemic stroke. Next, we summarize the mechanisms of action of TFEB in the various pathological processes of ischemic stroke, its role in the neurovascular unit, and the current therapeutic approaches. Finally, we focus on potential directions for future TFEB research. The information presented here could contribute to new drug designs and clinical therapeutic approaches for ischemic stroke.

## 2 THE STRUCTURE OF TFEB

Other than TFEB, the MiT/TFE family also includes TFE3, TFEC, and MiT/TFE-related transcription factors (MITF) [19, 20], which are highly conserved in animal evolution [21]. Moreover, TFEB appears similar to the only MITF member in *Drosophila* [22] and HLH-30 in *Caenorhabditis elegans* [23]. This indicates that TFEB is the most important protein in the MiT/TFE family, which is essential for the survival of the organism.

**Fig. (1):**
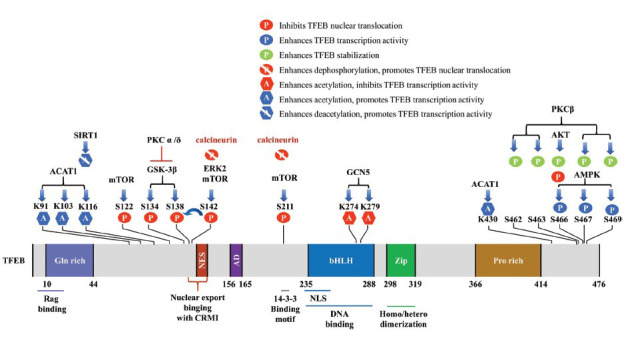
Structure of TFEB. TFEB is a protein consisting of 476 amino acid residues, mainly including a glutamic acid-rich domain (Gin-rich), nuclear export signal (NES), an acidic transcription activation domain (AD), bHLH-Zip structure, and proline-rich motifs (Pro-rich). Phosphorylation and acetylation are the most important post-translational modifications that regulate TFEB subcellular structure and transcription activity. The phosphorylated or acetylated TFEB sites of different proteins have been shown in the figure.

TFEB comprises 476 amino-acid residues and several major structural domains, including a glutamate-rich domain, an acidic transcriptional activation domain, a nuclear export signal (NES), a bHLH-Zip structure, and a proline-rich domain [24]. The bHLH-Zip region is required to form TFEB homodimers and heterodimers, which activate the expression of their target genes by binding to a palindromic DNA sequence (CACGTG, E-box) located in the proximal promoter of the target genes [20, 25, 26]. However, unlike other HLH leucine‐zipper transcription factors, MiT‐TFE proteins also bind the asymmetric DNA sequence (TCATGTG, M-box) [27]. Additionally, structural and biochemical evidence suggests that MiT‐TFE proteins may heterodimerize with one another but not with other members of the HLH/leucine‐zipper family due to an unusual three-residue shift in the leucine zipper register; moreover, the MiT-TFE homodimer has a marked kink in one of the two zipper helices that allow out-of-register assembly [28]. The NES is an evolutionarily conserved hydrophobic sequence located at the N-terminal of TFEB, specifically recognized by the nuclear export receptor CRM1, and facilitates the nuclear export of TFEB [29]. The transcriptional activation domain is conserved and present in all MiT/TFE families except for TFEC, which converts TFEC into a repressor of transcriptional activation [6, 30] (Fig. **1**).

## 3 REGULATION OF TFEB TRANSCRIPTIONAL ACTIVITY

Transcriptional regulation is an important process by which cells maintain cellular homeostasis and respond to environmental challenges. TFEB is a significant transcription factor sequestered in the cytoplasm in an inactivated form and transferred to the nucleus in an activated form where it can transcribe its target genes [31]. Therefore, the transcriptional activity of TFEB can be evaluated by monitoring the translocation of TFEB to the nucleus in response to multiple activating stimuli such as nutritional deprivation [9, 32], infection [33, 34], immune response [35], bacterial phagocytosis [36], mitochondrial dysfunction [37], physical exercise, [11] and endoplasmic reticulum stress [38]. Of these, nutrient deprivation-induced nuclear translocation is the most studied.

### 3.1 Phosphorylation and Dephosphorylation

Phosphorylation is a major post-translational modification regulating the activity of intracellular transcription factors [39]. At homeostasis, the activity of TFEB is inhibited by serine phosphorylation, resulting in sequestration in the cytosol. Upon stress application, TFEB is dephosphorylated to the active form and translated to the nucleus [40, 41]. TFEB interacts with the following kinases and phosphatases.

#### 3.1.1 mTORC1

The mammalian target of rapamycin complex 1 (mTORC1) is located at the crossroads of anabolism and catabolism, and the regulatory direction depends on the level of the intracellular energy supply [42]. TFEB cytoplasm/nuclear shuttling is driven by mTORC1-dependent multisite phosphorylation (Ser122, Ser142, and Ser211) [43]. mTORC1 phosphorylation of TFEB at Ser211 masks its nuclear localization signal and creates a high-affinity binding site for the 14-3-3 protein, leading to its retention in the cytosol [31, 43, 44]. However, dephosphorylation at Ser211 is not sufficient to localize TFEB exclusively to the nucleus since mTORC1-mediated regulation survives the mutation of serine 211 to alanine (S211A) [43]. Ser122 is directly phosphorylated by mTORC1. Its mutation to aspartate (S122D) inhibits nuclear localization of the S211A TFEB mutant. In contrast, the dual mutant (S122A and S211A) allows maximum nuclear enrichment in TFEB, which indicates that Ser122 and Ser211 dephosphorylation are crucial for TFEB nuclear localization [43]. TFEB Ser142 is another phosphorylation site for mTORC1, but its functional significance remains unclear [43]. These results suggest that mTORC1 is involved explicitly in controlling TFEB cytosolic retention. mTORC1, in turn, is regulated by the tuberous sclerosis complex 1 (TSC1) and 2 (TSC2) proteins, which is a process crucially involved in oxygen signaling [45, 46]. An abnormal increase in mTORC1 activity is observed under serum starvation in TSC1/TSC2-deficient cells, which can be mitigated by rapamycin treatment [40]. Furthermore, like rapamycin, raptor siRNA inhibits mTORC1 and retards TFEB mobility in TSC2-depleted HeLa cells [40]. However, the mechanism underlying the differential regulation of the subcellular localization of TFEB by mTORC1 in TSC1/TSC2-deficient cells remains unclear.

#### 3.1.2 AMPK, Akt, and ERK

AMP-activated protein kinase (AMPK), an intracellular energy sensor, phosphorylates TFEB at Ser466 and Ser467, activating it for transcription under conditions of nutrient restriction and pharmacological manipulation of mTORC1 [47, 48]. Protein kinase B (Akt) is essential for cell survival and apoptosis [49]. TFEB phosphorylation by Akt at Ser467 inhibits its nuclear translocation independent of mTORC1 [50]; in contrast, phosphorylation of the TFEB mutant S467A increases its stability, nuclear translocation, and transcriptional activity [50, 51]. Furthermore, the extracellular signal-regulated kinase (ERK) can phosphorylate TFEB at Ser142 and modulate its nuclear import [9, 52].

#### 3.1.3 GSK-3β and PKC

Glycogen synthase kinase-3β (GSK-3β) is crucial in regulating glucose metabolism and energy homeostasis [53]. Phosphorylation of TFEB at Ser134 and Ser138 by GSK-3β enhances TFEB binding to 14-3-3 proteins and its retention in the cytosol [54, 55]. Protein kinase C (PKC) activates TFEB and inactivates the ZKSCAN3 transcriptional repressor through two parallel signal cascades [56]. On the one hand, PKCα and PKCδ inactivate GSK-3β in a mTORC1-independent manner, which reverses the GSK-3β-induced TFEB inhibition and enhances its transcriptional activity. Notably, the activated PKC-GSK-3β cascade only targets TFEB and not TFE3 [56]. Further, activated PKCβ phosphorylates TFEB at Ser462, Ser463, Ser467, and Ser469, which enhances TFEB stability without affecting its subcellular localization [57]. On the other hand, ZKSCAN3 phosphorylation by PKC promotes its translocation from the nucleus and inactivation, which relieves the ZKSCAN3-induced transcriptional repression of TFEB [56].

#### 3.1.4 Calcineurin and Calcium Signaling

Calcium (Ca^2+^) is an important intracellular messenger that activates enzymes and transcription factors. Calcineurin activation by lysosomal Ca^2+^ dephosphorylates TFEB at Ser211 and Ser142, which promotes its nuclear translocation [5, 58, 59]. Furthermore, intracellular or extracellular Ca^2+^ promotes TFEB nuclear translocation through the Ca^2+^/ calmodulin-dependent protein kinase 2-AMPK-mTORC1 signaling pathway [58].

The nuclear export of TFEB is the limiting step of its subcellular localization [60]. The ERK-mTORC1-induced phosphorylation of TFEB at Ser142 primes it for GSK-3β-induced phosphorylation at Ser138, which activates the NES, for which both phosphorylations are necessary [29, 61]. The NES is specifically recognized by the export protein CRM1, which subsequently induces the nuclear export of TFEB [62]. The mechanisms underlying the relationship of TFEB Ser142 and Ser138 phosphorylation with nuclear export remain unclear. However, it has been speculated that Ser138 and Ser142 are involved in CRM1 binding to the NES site and facilitating TFEB nuclear export, given their proximity. The TFEB NES regulates carbon (glucose) and nitrogen (amino acid) availability by controlling the nuclear import-export cycle of TFEB [61].

### 3.2 Acetylation

Acetylation is an important post-translational modification that regulates TFEB transcriptional activity and subcellular localization [63]. Acetylation of TFEB at lysines K274 and K279 by the histone acetyltransferase GCN5 disrupts its dimerization, which prevents it from binding to the target gene promoter [64]. Conversely, TFEB deacetylation at K116 by the histone deacetylase SIRT1 enhances its transcriptional activity [65]. Although TFEB acetylation appears to inhibit its activity, there have been conflicting reports. Specifically, TFEB acetylation at K91, K103, K116, and K430 by the histone acetyltransferase ACAT1 enhances its transcriptional activity [12]; moreover, inhibition of the histone deacetylase HDAC6 to enhance TFEB acetylation induces its nuclear import and initiates the expression of its target gene [66]. These inconsistent reports could be attributed to different research backgrounds. Therefore, further research on the effect of acetylation on TFEB activity is warranted.

### 3.3 Ubiquitination

As previously mentioned, TFEB is a dimer when active [20, 25, 26]. In a nutrient‐rich environment, activated mTOR phosphorylates TFEB to form inactive homodimers of phosphorylated TFEB, which comprise the major pool of TFEB in the cytosol. Upon application of stress such as starvation and/or mTOR inhibition, TFEB is activated by dephosphorylation, leading to increased formation of active non-phosphorylated homodimers of TFEB and inactive heterodimers formed from phosphorylated and non-phosphorylated TFEB. Therefore, phospho-TFEB reduces TFEB activity and interferes with its nuclear translocation by forming heterodimers with otherwise active dephosphorylated TFEB. Hence, targeting phosphorylated TFEB for degradation is an important mechanism to enhance TFEB activity.

STIP1 homology and U-Box-containing protein 1 (STUB1) is a chaperone-dependent E3 ubiquitin ligase that promotes ubiquitin-mediated protein degradation and aids cellular recovery from stress [67]. Sha and his colleagues observed that during stress, STUB1 preferentially interacted with and ubiquitinated phosphorylated TFEB, thereby targeting it for proteasomal degradation [68]. As a result, non-phosphorylated TFEB was released from inactive heterodimers and incorporated into active, non-phosphorylated homodimers. These additional homodimers translocated to the nucleus and activated the target genes, including their gene, further increasing the level of active TFEB in the cell [69]. TFEB activity/level of non-phosphorylated TFEB is thus negatively correlated with the cellular level of phosphorylated TFEB, degrading inactive, phosphorylated TFEB and re-synthesizing new TFEB constituting an important cellular mechanism of responding to cellular stress conditions.

In addition, the proteasome and autophagy systems are two major cellular degradation systems that play essential roles in maintaining proteostasis. STUB1 mediates the degradation of TFEB in proteasomes. Since TFEB is a regulator of autophagy, its study has provided new insights into the interaction of the two degradation systems.

## 4 TFEB SENSES AND REGULATES ENERGY METABOLISM IN PATIENTS WITH ISCHEMIC STROKE

Impaired energy metabolism is a key pathological hallmark of ischemic stroke, and ischemia is one of the most important forms of energy deprivation [70]. When a stroke occurs, the transport of oxygen, glucose, amino acids, and other substrates of energy metabolism is severely limited. To survive, neurons change their glucose metabolism pathway from aerobic oxidation to anaerobic glycolysis to maintain adenosine triphosphate (ATP) production and, thus, homeostasis. However, compared with aerobic oxidation (glycolysis coupled to oxidative phosphorylation), which produces 38 ATP molecules per molecule of glucose, the anaerobic process (glycolysis alone) has only two ATP molecules per molecule of glucose [71]. This inefficient energy production pathway rapidly results in excess consumption over production and a decrease in intracellular ATP concentration [72]. It has been shown that during the first 5 minutes following AIS onset, blood flow in the ischemic core is reduced by more than 80%, and glucose and ATP levels decrease significantly [73]. The brain draws on its phosphocreatine stores to maintain ATP levels and restore energy homeostasis, which acts as a short-term energy reserve, allowing ATP regeneration from ADP in a reaction catalyzed by creatine kinase [74]. AMPK acts as an early sensor of energy deprivation for maintaining metabolic homeostasis and is activated by increases in the AMP/ATP or ADP/ATP ratios following AIS [75]. Activated AMPK also inhibits mTORC1 by directly phosphorylating TSC1/TSC2 to block anabolic pathways that consume ATP, such as fatty acid synthesis, gluconeogenesis, and protein synthesis [76, 77]. Inhibition of mTORC1 activity also dephosphorylates TFEB to enhance its nuclear translocation. On the other hand, AMPK directly phosphorylates TFEB to enhance its transcriptional activity, which promotes catabolic pathways that generate ATP, such as glucose uptake, anaerobic glycolysis, and fatty-acid oxidation [48]. Overall, these energy regulation measures maintain ATP concentrations at 15-30% of homeostasis levels in the ischemic core and 50% in the penumbra during the first 2 hours following AIS, which usually helps to meet the short-term energy needs of the brain [78, 79].

**Fig. (2):**
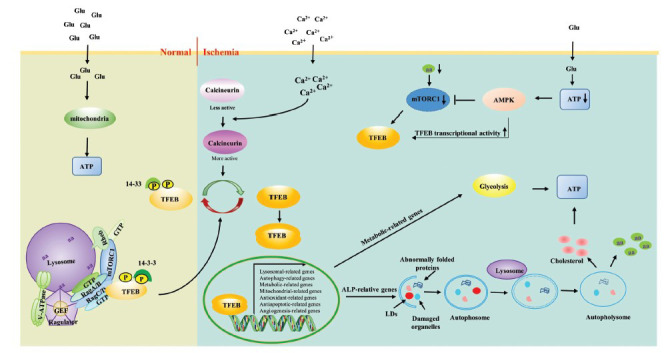
TFEB senses and regulates energy metabolism in patients with ischemic stroke. Under nutrient-rich conditions, cells metabolize glucose to produce sufficient ATP. In addition, the intracellular amino acid supply is sufficient, and v-ATPase interacts with the Ragulator to activate its guanine nucleotide exchange factor (GEF). The ragulator then recruits and assembles RagA-D into Rag A/B and Rag C/D heterodimers. The Rag GTPases heterodimers then interact directly and synchronously with the Rag binding region of TFEB and a subunit of mTORC1, and Raptor, respectively. Subsequently, with the help of growth factors, the small GTPase Rheb activates mTORC1 on the lysosome, and the activated mTORC1 phosphorylates TFEB at Ser211, leading to retention on the TFEB cell membrane. When a stroke occurs, impaired glucose and amino acid transport lead to reduced ATP production. AMPK, an early sensor of intracellular energy deprivation, is activated to survive. On the one hand, activated AMPK inhibits mTORC1 to block the anabolic pathway that consumes ATP. At the same time, the inactivation of mTORC1, together with the activation of specific phosphatases such as calmodulin, leads to the nuclear translocation of TFEB, which then induces the transcription of target genes to produce ATP to maintain short-term cellular energy needs. On the other hand, AMPK directly phosphorylates TFEB to enhance its transcriptional activity, thereby promoting the catabolic pathway that generates ATP.

Nutrient deficiencies such as declining amino acid levels can be sensed by mTORC1 independently of AMPK when cells are chronically energy-deprived, a process in which lysosomes play a rather complex role. Fluctuations in amino acid levels within the lysosomal lumen affect the conformation of vacuolar (H^+^)-ATPase (V-ATPase). Under intracellular amino acid-rich conditions, V-ATPase interacts with Ragulator, a pentagonal protein complex located on the outer lysosome surface, to activate the Ragulator guanine nucleotide exchange factor (GEF) [80]. Subsequently, Ragulator recruits GTP-bound Rag-related GTP-binding protein A (RRAGA, Rag A), RRAGB (Rag B), RRAGC (Rag C), and RRAGD (Rag D) to the lysosome and, through its GEF activity, facilitates the binding of Rag A or Rag B to Rag C or Rag D [81, 82]. Taken together, these ragulator interactions convey information regarding amino acid availability *via* the nucleotide status of the small GTPase Rags [83]. Furthermore, mTORC1 monitors cellular nutrient levels and has a complex and cell context-dependent regulatory effect on TFEB [40, 41]. Under nutrient-rich conditions, the heterodimeric Rag GTPases directly interact with a Rag-binding region of TFEB and with a mTORC1 subunit called Raptor, respectively, which allows spatiotemporal coordination between TFEB and mTORC1 [82, 84]. Subsequently, with the assistance of growth factors, the small GTPase Rheb activates lysosomal mTORC1, leading to TFEB phosphorylation at Ser211 and, consequently, its cytosolic sequestration [85]. Conversely, under nutrient-deprived conditions following ischemic stroke, the nucleotide states of the Rags are altered so that they cannot bind to mTORC1 or TFEB. Subsequently, mTORC1 is inactivated, which reverses TFEB repression by mTORC1-mediated phosphorylation. Consequently, this leads to TFEB nuclear translocation and induces the transcriptional expression of its target genes, including lysosomal, autophagy, and metabolism-related genes (Fig. **2**) [31, 44, 86, 87]. Notably, under starvation conditions, abnormal Rag GTPase heterodimers recruit another set of proteins to the lysosome, including folliculin and TSC. The TSC complex acts as a GTPase-activating protein to inhibit Rheb activity, further facilitating mTORC1 inactivation. Folliculin has GTPase activity toward Rag C/D, which contributes to rapid mTORC1 re-activation under re-fed conditions [88, 89]. TFEB regulates folliculin and its interacting proteins FNIP1 and FNIP2, which indicates that TFEB facilitates the cellular response to starvation and prepares itself for an efficient transition to nutrient-enriched conditions [86].

These interactions show that TFEB plays an important role in balancing the cellular energy budget by sensing cellular ATP and nutrient levels (glucose and amino acids) in an AMPK- and mTORC1-dependent manner. Downstream from AMPK and mTORC1, TFEB is a key effector, inducing transcriptional expression of autophagy-related genes, thus preventing the accumulation of damaged proteins and organelles and promoting the recycling of building blocks such as fatty acids and amino acids, which are critical for maintaining ATP levels and the synthesis of essential survival components [90]. In addition, TFEB upregulates the expression of genes involved in lipid and glucose metabolism, producing ATP to maintain energy homeostasis [91, 92]. Overall, these findings converge on identifying TFEB as an important player in sensing and regulating energy levels *in vivo*.

## 5 TFEB REGULATES PATHOPHYSIOLOGICAL PROCESSES IN ISCHEMIC STROKE

The pathogenesis of ischemic stroke involves multiple factors, including dysfunction of the autophagy-lysosome pathway (ALP), metabolic disorders, mitochondrial dysfunction, oxidative stress, neuroinflammation, apoptosis, and disorders of angiogenesis, which are the primary reason for the failure of current single-target neuroprotective therapies [93, 94]. TFEB induces the transcriptional expression of various genes that may affect the multiple pathological processes of AIS. Therefore, an exploration of the mitigating effects of TFEB in AIS follows.

### 5.1 Amelioration of ALP Dysfunction

The ALP, a catabolic pathway for cellular self-protection, is often activated by AIS. Under ischemic conditions, proper autophagy activation contributes to maintaining cellular homeostasis and survival, while excessive or prolonged activation triggers neuronal death [95]. Neither up-regulation nor inhibition of autophagy has been shown to ameliorate ischemic injury [96], which indicates that ALP dysfunction is a promising target for stroke treatment.

Lysosomes are the terminal organelles of autophagy and maintain protein and cellular homeostasis by degrading damaged and misfolded proteins [97]. Lysosomal dysfunction is an important physiological process in AIS that leads to neuronal synaptic impairment or death [93, 98-100], while restoring lysosomal function has a neuroprotective effect in AIS [100, 101]. TFEB, a major transcriptional regulator of lysosomal biogenesis, directly binds to CLEAR elements (10-base E-box-like palindromic sequences located in promoters) and induces the expression of various genes, including those for lysosomal biogenesis, lysosomal hydrolases, and lysosomal membrane proteins [7, 102]. Additionally, TFEB controls the expression of numerous autophagic genes and induces autophagosome-lysosome fusion and the biogenesis of autophagosomes [9]. Several drugs that target TFEB can rescue ALP dysfunction and reduce cerebral ischemic damage [93, 98, 100, 103, 104].

### 5.2 Enhancement of Cellular Metabolism

Metabolism, which comprises numerous interconnected cellular pathways of anabolism and catabolism, provides cells with the energy required to perform physiological functions [105]. Cells protect themselves from ischemic injury by inhibiting metabolism and promoting catabolism. Therefore, metabolic dysfunction is a potential target for ischemic stroke treatment, and TFEB is an emerging metabolic regulator that can reprogram glucose and lipid metabolism [106].

Glucose is the primary energy-supplying substrate for neurons. Under physiological conditions, glucose enters cells through specific glucose transporters. It is almost entirely oxidized to carbon dioxide and water with concurrent production of ATP to meet the cellular energy demand. Glucose figures in various metabolic pathways of the brain, including glycolysis, the pentose phosphate pathway, and (in astrocytes only) glycogenesis [107, 108]. Following AIS, neurons depend highly on an uninterrupted ATP supply from glycolysis [109]. However, under these conditions, a cascade of reactions induced by DNA damage inhibits intracellular glycolysis and the mitochondrial pyruvate supply, further exacerbating neuronal damage [110]. TFEB can ameliorate glucose metabolism to maintain ATP homeostasis following AIS. First, TFEB promotes glucose uptake and metabolism by upregulating the expression of insulin receptor substrates 1 and 2 [111]. Then TFEB induces the expression of glycolytic genes, which promotes ATP production [92].

Lipids are another major energy source for living organisms. Under nutrient-deprived conditions, lipolysis is increased and modulated by numerous transcription factors, which allows rapid adaptation to the situation, maintains basic homeostasis, and supports energy requirements [112]. TFEB is among the transcription factors that efficaciously regulate lipid metabolism; specifically, it controls lipid transport and lipolysis mediated by lysosomes and fatty-acid oxidation [113, 114]. The TFEB-CLEAR pathway upregulates the ALP and is crucial in lipid metabolism, pivotal in the cell’s adaptation to environmental changes [115]. When nutrients are scarce, cytoplasmic lipid droplets, which represent a major intracellular energy store, become encapsulated in a bilayer membrane structure to form autophagosomes that fuse with lysosomes and are degraded by acidic lipases to produce free cholesterol and fatty acids [112, 116]. Subsequently, fatty acids are effluxed and undergo re-uptake into mitochondria, where they undergo β-oxidation to yield energy or ketone bodies that support cellular energy requirements [112, 116]. In this connection, TFEB is crucial in maintaining mitochondrial homeostasis by promoting the β-oxidation of fatty acids in mitochondria and peroxisomes [10, 90]. The TFEB-CLEAR pathway is also involved in lipid catabolism mediated by phosphatidylinositol-5-phosphate 4-kinases, which are required for autophagosome-lysosome fusion [117]. Additionally, TFEB regulates lipid transport by influencing the expression of the cell surface protein CD36, which is responsible for fatty acid uptake, and the efflux protein ATP-binding cassette transporter A1 [118]. TFEB is also involved in regulating peroxisome proliferator-activated receptor-γ coactivator-1α (PGC-1α) and peroxisome proliferator-activated receptor α, which are the major lipid metabolism regulators [91]. Further, TFEB is involved in store-operated, calcium entry-controlled lipid metabolism, lipophagy induction, and fatty acid mobilization from lipid droplets [119, 120]. TFEB is therefore involved in regulating the entire process of lipid transport and catabolism.

### 5.3 Amelioration of Mitochondrial Dysfunction

Since the brain is one of the most energy-demanding organs, it depends on the mitochondria to provide an effective energy source to maintain its normal functions. Mitochondrial disruption is a key determinant of the degree of ischemic brain injury [121]. When AIS occurs, inadequate delivery of oxygen and glucose inhibits mitochondrial respiration, which is reduced by 45-60% in the active infarct core and by 15-40% in the ischemic penumbra 2 hours following ischemia, leading to reduced ATP production, intracellular calcium ion accumulation, and mitochondrial depolarization [122]. Succinate, a metabolite of the citric acid cycle, dramatically accumulates during ischemia and is rapidly oxidized upon reperfusion; oxidized succinate then drives the production of reactive oxygen species (ROS) at complex I by reverse electron transport, which further exacerbates mitochondrial functional impairment and further reduces ATP production in a vicious cycle [123]. To survive, cells employ mitochondrial quality control, including mitochondrial fission and fusion, mitophagy, and mitochondrial biogenesis, to maintain the homeostasis of intracellular mitochondrial quality and quantity [124].

Mitochondrial fission and fusion are the processes whereby mildly damaged mitochondria compensate for their loss of function by fusing with other healthy mitochondria or by fissioning to produce healthy daughter mitochondria or eliminate harmful components. The delicate dynamic balance between fission and fusion is critical for maintaining optimal mitochondrial function and meeting specific metabolic and energetic cellular demands following ischemic brain injury [125]. Drp1 (dynamin-related protein 1), which mediates mitochondrial fission, and Mfn 1 (mitofusin 1) and Opa 1 (optic atrophy 1), which mediate mitochondrial fusion, are all GTPases and thus have the ability to regulate the phosphorylation of TFEB *via* mTORC1 [124, 126]. Following cerebral ischemia onset, the balance between mitochondrial fission and fusion is disrupted, and TFEB activity is altered; therefore, it is reasonable to speculate that TFEB is involved in maintaining the balance between mitochondrial fission and fusion.

Mitophagy is a lysosome-dependent pathway for the self-clearance of damaged mitochondria that cannot be recovered by fission or fusion and involves pathways dependent on and independent of PTEN-induced putative kinase 1 (PINK1) and Parkin RBR E3 ubiquitin-protein ligase (PRKN). PINK1-PRKN-dependent mitophagy is the best-studied pathway in mammalian cells [127]. Mitophagy and mitochondrial biogenesis are in balance and control the quality and quantity of the mitochondria, and TFEB plays an important role in the balancing process. PINK1 is a Ser/Thr kinase. Following cerebral ischemia, mitochondrial depolarization leads to blocked cleavage of PINK1, which then recruits Parkin to damaged mitochondria to activate mitochondrial autophagy. In this process, PINK1 can activate TFEB directly or through a kinase cascade reaction, whereby PINK1 first phosphorylates Parkin, which then inactivates Rag GTPases and dissociates mTORC1 from TFEB, thereby reducing TFEB phosphorylation and allowing its translocation to the nucleus [37, 87]. Next, activated TFEB degrades damaged mitochondria *via* the ALP pathway and can simultaneously upregulate PGC-1α to promote mitochondrial biogenesis [128, 129].

Carbon monoxide (CO) is an endogenous gaseous transmitter that promotes cellular homeostasis and cytoprotection by regulating mitochondrial function and cellular metabolism. Endogenous CO is mainly produced catalytically by heme oxygenase [130]. Hypoxia-inducible factor-1 is a heterodimeric transcription factor that mediates the adaptive response to hypoxia. Its targets include genes involved in vasodilatory control, angiogenesis, erythropoiesis, cell proliferation, and energy metabolism, all of which may contribute to neuronal cell recovery following cerebral ischemia and reperfusion [131]. When AIS occurs, hypoxia-inducible factor-1 expression is upregulated to mediate the transcriptional activation of heme oxygenase-1. This leads to increased production of endogenous CO and, consecutively, to TFEB nuclear translocation, thereby enhancing mitochondrial autophagy and mitochondrial biogenesis [131, 132].

Once damage exceeds the capacity of mitochondrial quality control, the mitochondria translate these danger signals into cell-death decisions leading to processes such as mitochondria-mediated apoptosis; the role of TFEB in this process will be described in detail below.

### 5.4 Inhibition of Oxidative Stress

Oxidative stress is a major pathological process in ischemic stroke, manifesting as an imbalance between antioxidant activity and ROS production and exacerbating neuronal and blood-brain barrier (BBB) impairments [133, 134]. Therefore, oxidative stress is another important therapeutic target for ischemic stroke.

The TFEB-mediated regulation of oxidative stress involves multiple pathways. Following ischemic stroke, mitochondrial damage triggers a vicious cycle of ROS-injury-ROS that causes uncontrolled ROS production, which is among the leading causes of secondary damage [133, 134]. Offsetting negative feedback involves TFEB oxidation by increased ROS levels at cysteine 211 and its subsequent translocation to the nucleus [135]. Subsequently, TFEB mediates the ALP, which removes damaged mitochondria to break the ROS cycle and inhibit ROS production [135-137]. This process is also known as the type-II antioxidant response.

The nuclear erythroid 2-related factor 2 (encoded by *NFE2L2*) protects against oxidative and electrophilic stress. TFEB directly induces *NFE2L2* expression, promoting detoxification and an antioxidant response *via* the NFE2L2-ARE pathway [138, 139], also known as the type-I antioxidant response. These findings suggest that TFEB may protect against ischemic stroke by promoting type-I and type-II antioxidant responses.

### 5.5 Inhibition of Neuroinflammation

Neuroinflammation is a major pathological process in several acute and chronic brain diseases [140]. Following ischemic stroke, BBB disruption occurs, followed by infiltration of immune cells from the peripheral circulation into the ischemic area. Moreover, resident astrocytes and microglia in the central nervous system (CNS) are activated to release inflammatory mediators, chemokines, and complement proteins, further aggravating brain injury [141, 142]. Therefore, neuroinflammation following ischemic stroke also induces secondary injury [143]. Nuclear factor-κB (NF-κB) is a classical pro-inflammatory transcription factor that regulates the release of pro-inflammatory mediators, including cytokines, chemokines, and adhesion molecules [144]. TFEB downregulates NF-κB expression and inhibits NF-κB activity, suppressing the inflammatory response [145, 146]. Inflammasome activation is a major inflammatory pathway involved in the progression of inflammatory diseases. The pyrin domain-containing 3 protein (NLRP3), the best-studied inflammatory complex, is activated in AIS [147]. TFEB signaling negatively regulates the NLRP3 inflammasome through the following pathways [148]: 1) removing impaired mitochondria and reducing ROS production, which suppresses inflammasome activation [149]; 2) increasing lysosome-associated membrane protein type 2A expression, which binds to NLRP3 and promotes its degradation through chaperone-mediated autophagy [150]; and 3) enhancing p62-dependent degradation *via* chaperone-mediated autophagy [151]. Furthermore, the ALP promotes the degradation of pro-interleukin 1β, a product of the NLRP3 inflammasome [152].

### 5.6 Inhibition of Apoptosis

Apoptosis is an autonomous programmed cell death pathway that cells initiate to maintain the stability of the organism when they undergo continuous stress and fail to reach homeostasis. The process of apoptosis has been observed in neurons following ischemic stroke and involves cell shrinkage and cytoplasmatic condensation, followed by nuclear membrane breakdown and the formation of apoptotic bodies [153]. However, in neurons of the ischemic penumbra, it seems that apoptosis may be reversible, indicating that apoptosis is a potential therapeutic target in ischemic stroke [154].

Neuronal apoptosis following ischemic stroke involves both intrinsic and extrinsic pathways. When a stroke occurs, a dramatic drop in ATP levels in the neuron impairs the maintenance of ion gradients. It triggers a cascade reaction in which calcium ions accumulate in the neuron, which then disrupts the mitochondrial membrane and releases cytochrome C (Cytc) or apoptosis-inducing factor (AIF) [72]. After entering the cytosol, Cytc complexes form an apoptosome with apoptotic protein-activating factor-1 and procaspase-9, activating effector caspases such as caspase-3 [155]. Unlike Cytc, AIF can quickly translocate to the nucleus and mediate significant DNA fragmentation, leading to caspase-independent cell death [156]. Damaged DNA acts directly on mitochondria through the phosphorylation of p53 or the translocation of nucleophosmin, further amplifying the mitochondria-dependent apoptotic effect [157]. When blood flow is restored following cerebral ischemia, large amounts of ROS are produced, which may damage DNA and mitochondrial membranes and release Cytc to trigger apoptosis. This may be one of the causes of neuronal ischemia/reperfusion injury [158]. In parallel, glial cells and immune cells become activated during cerebral ischemia and release multiple cytokines that bind to “death receptors” and trigger neuronal apoptosis through extrinsic pathways. These cytokines include interleukin 1β, chemokines, tumor necrosis factor, and Fas ligand. Death receptors include tumor necrosis factor-related apoptosis-inducing ligand receptors [72, 159]. Extrinsic apoptosis can occur either independently or synergistically with the mitochondrial pathway. Following ischemic stroke, binding the death ligand to the receptor triggers a cascade reaction that sequentially activates caspase-8 and the downstream effector caspase-3 [160, 161] or mediates apoptosis *via* a mitochondria-dependent pathway [162].

The regulation of apoptosis by TFEB is sophisticated. Under nutrient-rich conditions, TFEB can selectively enhance FasL-mediated apoptosis by upregulating the basal autophagic capacity of cells, allowing them to maximize their growth [163]. Under prolonged stress conditions such as endoplasmic reticulum stress or prolonged starvation, activation of TFEB may increase the expression of pro-apoptotic factors that activate transcriptional factor 4 and inhibit the expression of anti-apoptotic factor B-cell lymphoma/leukemia-2 (Bcl-2). These changes induce mitochondria-dependent apoptosis to maintain environmental homeostasis [38]. In contrast, TFEB can exert anti-apoptotic effects by upregulating the apoptosis inhibitor Bcl-2 and decreasing the expression and activity of caspase-3 [164]. Following cerebral ischemia, TFEB can also regulate neuronal apoptosis *via* several indirect pathways, for example, promoting catabolic pathways to maintain ATP stability, thus reducing intracellular calcium ion accumulation; inhibiting ROS production; maintaining mitochondrial homeostasis; inhibiting the release of pro-inflammatory factors. Thus, TFEB is a key regulator in cellular decision-making for apoptosis, and its direction of action depends on the ability of the cell to maintain its own homeostasis in the environment in which it is placed.

### 5.7 Stimulation of Endothelial Cells and Angiogenesis

AIS is caused by a sudden occlusion of blood vessels, which leads to ischemia, hypoxia, and brain-tissue death. Patients with abundant and dense vascular collateral circulation are at low risk of stroke and have a relatively good prognosis if it does occur, which suggests that promoting angiogenesis in the ischemic region is crucial for neural repair [165]. TFEB-deficient mice died after 9.5-10.5 days of embryonic development due to severe underdevelopment of the placental vasculature [166]. Furthermore, mice with endothelium-specific TFEB overexpression showed ameliorated blood perfusion. They increased capillary density compared with wild-type, while endothelium-specific TFEB-knockout mice showed diminished blood flow restoration following reperfusion [12, 167]. These findings demonstrate that TFEB is crucial for promoting angiogenesis to alleviate ischemic injury.

**Fig. (3):**
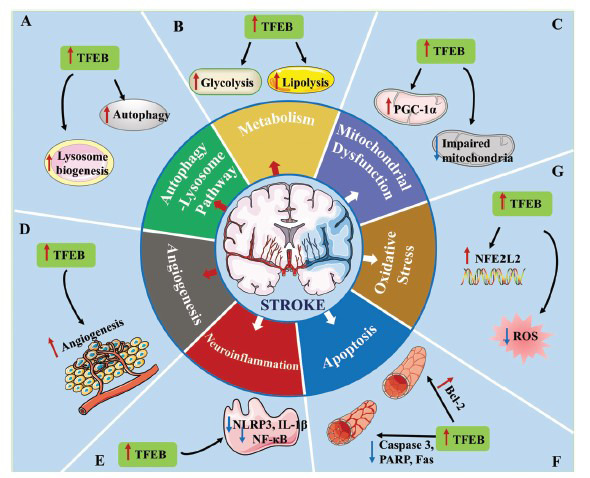
Roles of TFEB in ischemic stroke. (**A**) TFEB promotes autophagy and lysosome biogenesis against ischemic stroke. (**B**) TFEB enhances glycolysis and lipolysis protection against ischemic stroke. (**C**) TFEB ameliorates mitochondrial dysfunction against ischemic stroke; (**D**) TFEB promotes angiogenesis. (**E**) TFEB inhibits neuroinflammation in ischemic stroke. (**F**) TFEB inhibits apoptosis in ischemic stroke. (**G**) TFEB inhibits oxidative stress and promotes antioxidant properties in ischemic stroke. In short, TFEB exerts neuroprotective effects by regulating the mechanisms above.

Taken together, the research results on the regulatory mechanisms of TFEB sufficiently demonstrate that it is a good therapeutic target for ischemic stroke treatment (Fig. **3**).

### 5.8 Role of TFEB in the Neurovascular Unit (NVU)

**Fig. (4):**
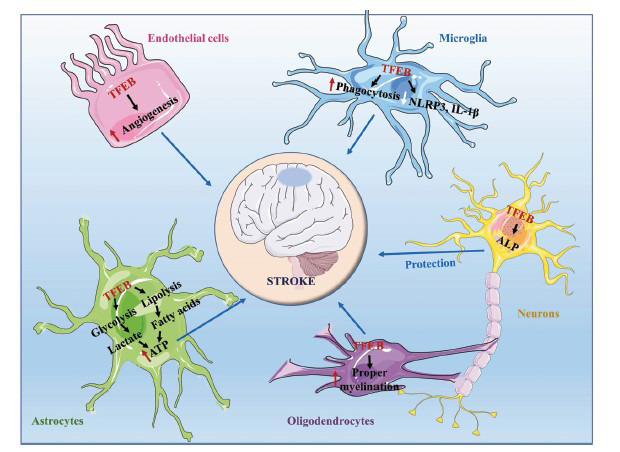
Protective roles of TFEB in the NVU under the ischemic stroke. NVU comprises neurons, astrocytes, microglia, oligodendrocytes, endothelial cells, smooth muscle cells, pericytes, and extracellular matrix. Under the ischemic condition, activated TFEB initiates a series of protective mechanisms in the NVU. TFEB improves the neuronal autophagy-lysosome pathway against ischemic stimuli. In astrocytes, TFEB enhances the catabolism of glycogen and glucose to produce ATP, while astrocytes do not utilize the metabolite of glucose, lactate. Besides, TFEB enhances the catabolism of Lipid droplets and Lipids to produce ATP. The remaining lactate and ATP are transported into neurons to maintain energy homeostasis. In microglia, TFEB enhances phagocytosis and inhibits the release of pro-inflammatory mediators. In oligodendrocytes, TFEB improves myelination and contributes to neuronal repair. In endothelial cells, TFEB enhances angiogenesis and eventually improves microcirculation.

The NVU comprises neurons, glial cells, endothelial cells, smooth muscle cells, pericytes, and an extracellular matrix. Its integrity is crucial for maintaining homeostasis within the brain microenvironment, regulating cerebral blood flow, and promoting neuro repair during AIS [168-170]. TFEB is highly expressed in the CNS and is crucially involved in maintaining the integrity of the NVU. Neurons form the core of the NVU and are very vulnerable to hypoxia. Neuron-targeted TFEB rescues ALP dysfunction, promotes synaptic plasticity, and alleviates ischemic injury [93, 98, 103]. Astrocytes are the major CNS components crucial in maintaining synaptic transmission and neuronal health in homeostasis [171]. Under normal conditions, astrocytes take up glucose from the vasculature and convert it to glycogen, a storage substance that can be degraded to provide metabolic support to adjacent neurons during ischemic stroke [172, 173]. TFEB enhances the catabolism of glycogen and lipid droplets in astrocytes to produce ATP [92], which is transported to neurons to maintain energy homeostasis [113, 114, 175]. Additionally, TFEB enhances the astrocytic support of neurons by upregulating lysosomal biogenesis and promoting the expression of brain-derived neurotrophic factors [174]. In addition, TFEB inhibits the release of pro-inflammatory cytokines from astrocytes [175]. Microglia are the resident immune cells of the CNS, and microglial-induced neuroinflammation causes secondary brain injury in AIS. TFEB signaling promotes microglial activation, ameliorates microglial phagocytic capacity, and reduces the secretion of pro-inflammatory mediators by upregulating lysosomal biogenesis [150, 176]. CNS functions depend on appropriate myelination for insulation and vital trophic support for axons [177]. Oligodendrocytes (OLs), which are involved in CNS myelination, are sensitive to ischemic damage [178]. Pre-myelinating OLs (pre-OLs) show high TFEB expression during the early postnatal period. Moreover, TFEB knockout in OL lineage cells causes ectopic survival of pre-OLs, which leads to aberrant myelination in brain regions that normally remain unmyelinated during development [179]. Conversely, continuous TFEB expression in myelinating OLs induces pro-apoptotic transcription *via* TP53 with subsequent activation of Bax/Bak-dependent programmed cell death, which is essential for selective OL elimination during development to ensure the spatiotemporal specificity of CNS myelination [179, 180]. Endothelial cells regulate vascular tone by producing vasoactive factors and maintain vascular permeability and the integrity of the NVU, smooth muscle cells, and pericytes [181]. The mechanism underlying TFEB-promoted angiogenesis has been previously described (Fig. **4**). Future studies should evaluate the actions of TFEB in NVUs in ischemic stroke; moreover, reliable clinical studies are warranted to validate and further explore the function of TFEB in human-derived cells.

## 6 THERAPEUTIC RESEARCH

Pharmacotherapy and physiotherapy are currently the main treatment strategies for ischemic stroke and may exert their AIS neuroprotective effects through TFEB signaling.

### 6.1 Pharmacotherapy

Pharmacotherapy is a classical and popular treatment, given its extensive approval and preference by patients. We review the literature and present the medicines and molecules that have been shown to modulate TFEB with statistical significance as follows, hoping to provide a basis for future drug development for AIS.

Multiple known clinical pharmacological interventions modulate TFEB in stroke patients. Hypoglycemic and hypolipidemic treatments are effective secondary preventative interventions for ischemic stroke, significantly reducing the risk of additional strokes and improving post-stroke outcomes. For example, the hypolipidemic drug fenofibrate promotes the release of lysosomal calcium ions and activates the calcineurin and CaMKKβ-AMPK pathways, subsequently promoting TFEB and TFE3 dephosphorylation and their nuclear translocation [182]. The hypoglycemic agent liraglutide is a glucagon-like peptide-1 receptor agonist whose receptor mimics TFEB to activate the autophagy-lysosome pathway [183]. The hypoglycemic agent metformin ameliorates autophagic flux and ameliorates ischemia/reperfusion injury by potentiating the AMPK-mTORC1-TFEB pathway [184]. Furthermore, antiplatelet agents are critical for successfully managing patients undergoing various neuro-interventional treatments. Aspirin activates peroxisome proliferator-activated receptor alpha to upregulate TFEB and increase lysosomal biogenesis in brain cells [185]. A novel antiplatelet aggregation agent, cilostazol, inhibits apoptosis following ischemia/reperfusion injury by upregulating the nuclear expression of TFEB [186, 187].

Several long-established clinical drugs can modulate TFEB. Celastrol is a promising candidate for treating Alzheimer's disease, with the potential to activate TFEB and improve the ALP [188]. 2-Hydroxypropyl-β-cyclodextrin, a clinical agent used to enhance the solubility of drugs, activates TFEB and inhibits the formation and development of abdominal aortic aneurysms in mice [164].

Several natural herbal derivatives have been shown to promote the nuclear translocation of TFEB and transcriptional activity, enhancing the ALP in neurons and attenuating cerebral ischemic injury. These include pseudoginsenoside F11 [103], melibiose [104], tomatidine [100], trehalose [189], genistein [190], naringenin [191], and curcumin and its analogs [192, 193].

### 6.2 Physical Therapy

Electroacupuncture, a special form of acupuncture, is characterized by easy control, objective measurement, and standardization and has shown good therapeutic effects in animal and clinical stroke studies [194]. Pretreatment with electroacupuncture induces cerebral ischemic tolerance [195], while the main treatment significantly promotes angiogenesis in the ischemic penumbra of rats [196]. Furthermore, electroacupuncture significantly enhances TFEB activity and improves ALP function in the brain [197], which indicates that it may ameliorate ischemic stroke *via* TFEB signaling.

Aerobic exercise is considered an effective strategy for maintaining physical and mental health. In patients with AIS, long-term aerobic exercise can reduce the morbidity and damage due to AIS by reducing risk factors such as hypertension, diabetes mellitus, and hyperlipidemia and enhancing cerebral ischemic tolerance [198]. Furthermore, aerobic exercise is a promising rehabilitation strategy because it can help restore blood circulation to the brain [199], re-establish the integrity of the BBB, ameliorate neurological function [200] and long-term prognosis, and reduce complications following ischemic events [201]. However, it is important to note that the timing of exercise rehabilitation of patients with AIS is important. Starting exercise within 24 hours following an ischemic event is detrimental because exercise interferes with the autoregulatory processes of the brain following AIS [202]. Conversely, 24 hours after the ischemic episode (in the subacute phase), the earlier exercise rehabilitation is started, the more beneficial it is [203].

The subacute phase of stroke relies on relatively slow molecular signaling pathways to determine neuronal apoptosis. During that phase, the fate of neurons depends on the interaction of pro- and anti-apoptotic molecules [204]. Prolonged aerobic exercise has been found to promote the nuclear translocation and transcriptional activation of TFEB in cortical areas through the AMPK-TFEB pathway, thereby activating lysosomal function, which is a possible mechanism by which exercise prevents AIS [204, 205]. Furthermore, recall that following AIS, TFEB can maintain ATP and amino acid homeostasis by promoting nutrient catabolism, reducing ROS production, maintaining mitochondrial homeostasis, and inhibiting the release of anti-inflammatory factors, thereby tending to prevent initiation of the internal/external apoptotic pathways. In addition, TFEB can exert anti-apoptotic effects by upregulating the apoptosis inhibitor Bcl-2 and decreasing the expression and activity of caspase-3 [164]. Therefore, TFEB may be a potential target for improving motor rehabilitation in ischemic stroke.

In addition, remote ischemic preconditioning, a valuable strategy for protection against AIS, can ameliorate post-stroke outcomes by targeting TFEB [190, 206].

These findings suggest that TFEB is a promising therapeutic drug candidate for ischemic stroke (Table **1**) [100, 103, 104, 164, 182-186, 189-191, 193, 197, 198, 204, 210-216].

## CONCLUDING REMARKS

Worldwide, stroke remains a major cause of mortality, showing high disability and morbidity rates that place enormous emotional and economic stress on individuals, families, and countries [217]. We have only recently begun to understand the contribution of TFEB to ischemic stroke. Moreover, the role of TFEB in cellular adaptation to ischemic stimuli is inextricably linked to its unique ability to regulate the ALP. Additionally, TFEB influences the expression of key genes involved in regulating mitochondrial function, metabolism, oxidative stress, apoptosis, and neuroinflammation. Given the broad functions of TFEB, elucidating the TFEB-mediated transcriptional network and investigating TFEB-targeting drugs may advance ischemic stroke treatment. However, several issues should be addressed. Future studies on TFEB are warranted, with the following aims: 1) to determine whether the TFEB activation observed in ischemic stroke is a causal factor or a compensatory response to disease progression and whether this depends on disease stage (*e.g*., acute *vs.* recovery phase); 2) to select small molecules or other druggable targets that could potentially be translated into human therapeutics through TFEB activation and to determine the potential effects of the extent and duration of TFEB activation when these selected targets are used for ischemic stroke treatment; 3) to evaluate TFEB-related targeting of the NVU in ischemic stroke, to determine whether the actions and responses of TFEB in ischemic stroke differ in a cell type-dependent manner, and to determine whether enhancing TFEB activity in different cell types can ameliorate the pathological status; 4) to translate reliable clinical research (*e.g*. randomized controlled trials) into TFEB-based therapeutics; and 5) to explore cerebral drug delivery systems involving TFEB signaling [218].

## Figures and Tables

**Table 1:** Therapeutic research of TFEB in ischemic stroke.

**Therapeutics**	**Compound**	**Chemical Nature and Source**	**TFEB Regulatory Mechanism**	**Potential Implication**	**Models/** **Subjects**	**Methods**	**Effects**
Pharmacotherapy	Pseudoginsenoside F11	An organic compounds is isolated from the aerial parts of *P. quinquefolium*.	Ameliorating the TFEB protein expression, nuclear translocation, and activity *via* calcineurin-TFEB pathway.	Ischemic stroke [104]	Male SD rats weighing 270-300 g	Treatment with Pseudoginsenoside F11 at 0.5 h after the onset of pMCAO and OGD	Pseudoginsenoside F11 ameliorates ALP dysfunction by activating calcineurin-mediated TFEB nuclear translocation in neurons during permanent cerebral ischemia.
Pharmacotherapy	Melibiose and trehalose	Trehalose, a natural disaccharide primarily extracted from *Selaginella tamariscina* Melibiose is an analogue of trehalose	Promoting TFEB nuclear translocation	Ischemic stroke [103, 189]	Male SD rats weighing 250-280 g	Pretreatment with melibiose for 7 days before MCAO/R	Melibiose confers neuroprotection against cerebral I/R injury by ameliorating ALP by facilitating TFEB nuclear translocation in neurons
Pharmacotherapy	Tomatidine	A gut microbiota-derived metabolite from unripe tomatoes	Increasing the expression and nucleus translocation of TFEB	Ischemic stroke [100]	Primary cortical neurons from C57 male mice.	Treatment with tomatidine at reinfusion after OGD	Tomatidine protects against ischemic neuronal injury by improving lysosomal function
-	Genistein	Genistein is an isoflavone first isolated from the brooming plant Dyer's Genista tinctoria L. and is widely distributed in the Fabaceae family	Control the expression and nucleus translocation of	Inherited metabolic diseases [190]	Human dermal fibroblasts.	Media containing concentrations of 30, 60 and 100 μm of genistein	Genistein modulates lysosomal metabolism and TFEB activation
Pharmacotherapy	Naringenin	Naringenin is a citrus-derived flavonoid naturally isolated from citrus fruits	Naringenin is directly bound to 14-3-3 epsilon blocked the TFEB-14-3-3 epsilon interaction, and therefore promoted TFEB nuclear translocation and activation	Abdominal aortic aneurysm flavonoid [191]	Angiotensin II-induced AAA Model in 16-week-old ApoE^−/−^ Mice	On postoperative day 2, naringin was received intragastrically (50 mg/kg/day)	Targeting macrophage TFEB-14-3-3 epsilon Interface by naringenin inhibits abdominal aortic aneurysm
Pharmacotherapy	Liraglutide	Liraglutide is an acylated glucagon-like peptide-1 analogue, is now used as a clinical hypoglycemic agent	Liraglutide is a glucagon-like peptide-1 receptor agonist, whose receptor mimics TFEB to activate the ALP	Hepatic steatosis [183], Ischemic stroke [207]	C57BL/6J mice weighing 23-28 g	Intraperitoneal liraglutide (400 μg/kg/day) for 4 weeks	Liraglutide attenuates hepatic steatosis *via* the GLP-1R-TFEB pathway, restoring ALP
Pharmacotherapy	Metformin	Metformin is currently the first-line drug treatment for type 2 diabetes	Promoting TFEB nuclear translocation by potentiating the AMPK-mTORC1-TFEB pathway	Survival of random-pattern skin flap [184], Ischemic stroke [208]	Male C57BL/6 mice weighing 20-30 g	Intraperitoneal metformin (200 mg/kg/day) from 7 days before operation until the mice were euthanized	Metformin promotes the survival of random-pattern skin flaps by inducing autophagy *via* the TFEB signaling pathway
Pharmacotherapy	Fenofibrate	Fenofibrate is FDA approved to treat patients with hypertriglyceridemia, primary hypercholesterolemia, or mixed dyslipidemia	Promotes TFEB nuclear translocation and TFEB activity *via* the calcium- calcineurin -TFEB/CaMKKβ-AMPK-TFEB pathway	NAFLD [182], Ischemic stroke [209]	Mice	Treatment with fenofibrate for 4 weeks	Fenofibrate reduces hepatic fat accumulation through the upregulation of TFEB-mediated lipophagy
Pharmacotherapy	Cilostazol	Cilostazol is a novel antiplatelet aggregation agent indicated for secondary prevention in patients with a history of transient ischemic attacks or non-cardioembolic ischaemic stroke	Upregulating TFEB nuclear translocation	Myocardial I/R injury [186], Ischemic stroke [210]	Male SD rats weighing 180-220 g	Intragastrical cilostazol (20 mg/kg) 30 min before I/R injury operation	Cilostazol protects against myocardial I/R injury by activating TFEB
Pharmacotherapy	Aspirin	Aspirinis an antiplatelet medicine that is effective for secondary prevention after minor ischemic stroke or transient ischemic attack	Aspirin activates peroxisome proliferator-activated receptor alpha to upregulate TFEB mRNA expression and TFEB nuclear translocation	Alzheimer's disease [185], Ischemic stroke [211]	Six to seven months old male and female 5xFAD mice and primary astrocytes	Treatment with aspirin (2 mg/kg body weight/d) *via* gavage for 30 d. Astrocytes were treated with aspirin for 4 h	Aspirin activates peroxisome proliferator-activated receptor alpha to upregulate TFEB and increase lysosomal biogenesis in brain cells
Pharmacotherapy	Celastrol	Celastrol is a pentacyclic triterpenoid extracted from *Tripterygium wilfordii*	Promotes TFEB nuclear translocation *via* mTORC1-TFEB pathway.	Alzheimer disease [188].	1.5-Month-old male and female homozygous human P301S Tau transgenic mice	Treated daily with celastrol at the concentrations of 1 and 2 mg/kg/day or vehicle for 2.5 months	Celastrol enhances TFEB-mediated autophagy
Pharmacotherapy	2-hydroxy-propyl-β-cyclodextrin	Hydroxypropyl-β-cyclodextrin is a hydroxyalkylated derivative of β-cyclodextrin, which has better water solubility and more significant solubilizing effect on insoluble drugs, and is currently used in clinical formulations to improve the solubility of drugs	Promotes TFEB nuclear translocation *via* mTORC1-TFEB pathway	Abdominal aortic aneurysm [164]	Vascular smooth muscle cell-selective Tfeb knockout C57BL/6J mice	Hyperlipidemia by AAV-PCSK9 D337Y and western diet, and infused with AngII (1,500 ng/kg/min) for another 4 weeks. Saline (vehicle control) or HPβCD (2 g/kg) were administered (i.p.) twice a week, starting one day before AngII infusion. starting one day before angiotensin II infusion	2-hydroxypropyl-β-cyclodextrin prevents abdominal aortic aneurysm *via* activation of vascular smooth muscle cell TFEB
Pharmacotherapy	Curcumin analog-C1	Curcumin is a natural medicine containing phenol and quinone groups that extracted from the rhizomes of Zingiberaceae and Araceae plants	Compound C1 specifically binds to TFEB at the N terminus and promotes TFEB nuclear translocation without inhibiting MTOR activity. TFEB nuclear translocation	Ischemic stroke [193]	Male SD rats weighing 350-400 g	Oral administration by gavage with C1 (10 mg/kg and 25 mg/kg per day) for 24 h	Compound C1 specifically binds to TFEB at the N terminus and promotes TFEB nuclear translocation without inhibiting mTOR activity
Pharmacotherapy	Digoxin, a clinically approved drug; ikarugamycin, a marine-derived natural product; alexidine dihydrochlorid, a synthetic compound.	A nanotechnology-enabled high-throughput screen to identify small-molecule compound	Activate TFEB *via* calcium- calcineurin/ CaMKKβ-AMPK-TFEB pathway	Metabolic syndrome [212], Ischemic stroke [213]	Four to six weeks old male C57BL/6 J mice	High-fat diet containing 60% fat	High-fat diet attenuates metabolic disorders in mice and extends the lifespan of *C. elegans*
RIPC	RIPC	-	Promoting TFEB protein expression and nuclear translocation	Chronic cerebral ischemia [214]	Male Wistar rats weighing 250-300 g	RIPC treatment (stopping femoral artery blood flow for 5 min, and then reperfusion for 5 min) 4 times a day for 2 weeks	RIPC attenuates damage in rats with chronic cerebral ischemia by upregulating the autophagolysosome pathway *via* TFEB activation
Electroacupuncture	Electroacupuncture	-	Promoting TFEB protein expressionandnuclear translocation	Alzheimer’s disease [197], Ischemic stroke [215]	Male 5xFAD mice	Electroacupuncture treatment 5 times per week for 4 weeks	Electroacupuncture ameliorates beta-amyloid pathology and cognitive impairment in Alzheimer’s disease *via* a novel mechanism involving the activation of TFEB
Aerobic exercise	Exercise [204]	-	Promoting TFEB nuclear translocation *via* AMPK-TFEB pathway	Ischemic stroke [216]	Mice	Wheel running at 18 rpm (14:00-17:00), 5 d/wk, for 8 weeks	Exercise activates the lysosomal function in the brain through the TFEB pathway

**^a^Abbreviations:** ALP, autophagy-lysosomal pathway; I/R, Ischemia/Reperfusion; MCAO/R, middle cerebral artery occlusion/reperfusion; NAFLD, nonalcoholic fatty liver disease; OGD, oxygen-glucose deprivation; pMCAO, permanent middle cerebral artery occlusion; RIPC, remote ischemic postconditioning; SD, Sprague-Dawley; TFEB, transcription factor EB.

## LIST OF ABBREVIATIONS

AIF: Apoptosis-inducing Factor
AIS: Acute Ischemic Stroke
Akt: Protein Kinase B
ALP: Autophagy-lysosome Pathway
AMPK: AMP-activated Protein Kinase
ATP: Adenosine Triphosphate
BBB: Blood-brain Barrier
Bcl-2: B-cell Lymphoma/leukemia-2
bHLH-Zip: Basic Helix-loop-helix Leucine-zipper
Ca^2+^: Calcium
CNS: Central Nervous System
CO: Carbon Monoxide
Cytc: Cytochrome C
ERK: Extracellular Signal-regulated Kinase
GEF: Guanine Nucleotide Exchange Factor
GSK-3β: Glycogen Synthase Kinase-3β
MiT/TFE: Microphthalmia
MITF: MiT/TFE-associated Transcription Factor
mTORC1: Mammalian Target of Rapamycin Complex 1
NES: Nuclear Export Signal
NF-κB: Nuclear Factor-κB
NLRP3: Pyrin Domain-containing 3 Protein
NVU: Neurovascular Unit
OLs: Oligodendrocytes
PRKN: Parkin RBR E3 Ubiquitin-protein Ligase
PGC-1α: Peroxisome Proliferator-activated Receptor-γ Coactivator-1α
PINK1: PTEN-induced Putative Kinase 1
PKC: Protein Kinase C
pre-OLs: pre-myelinating OLs
ROS: Reactive Oxygen Species
RRAGA: Rag-related GTP Binding-protein A
STUB1: STIP1 Homology and U-Box-containing Protein 1
TFEB: Transcription Factor EB
TSC: Tuberous Sclerosis Complex
V-ATPase: Vacuolar (H^+^)-ATPase
